# Failure of Nasal Defect Closures Following the Use of Continuous Positive Airway Pressure

**DOI:** 10.7759/cureus.36084

**Published:** 2023-03-13

**Authors:** Yelena Dokic, Emily Powell, John Kohorst, Ikue Shimizu

**Affiliations:** 1 Dermatology, Baylor College of Medicine, Houston, USA; 2 Dermatology, Wasatch Dermatology, South Ogden, USA; 3 Dermatology, Mayo Clinic Health System Franciscan Healthcare, La Crosse, USA

**Keywords:** mohs micrographic surgery, wound dehiscence, flap necrosis, mohs surgery, continuous positive airway pressure (cpap)

## Abstract

In this paper, we report a case series of three patients who developed nasal tip necrosis following Mohs micrographic surgery (MMS), complicated by the concomitant use of a continuous positive airway pressure (CPAP) machine for sleep apnea.

## Introduction

Postoperative factors, such as improper wound care, infection, hematoma formation, and smoking, are known to increase the risk of failure of surgical wound healing, as they contribute to ischemia, thus any other factor that reduces local blood supply can result in necrosis [1-3]. Herein, we present, for the first time in the literature, a case series of three patients who developed nasal tip necrosis following Mohs micrographic surgery (MMS), complicated by the concomitant use of a continuous positive airway pressure (CPAP) machine for sleep apnea.

## Case presentation

Patient 1

A 48-year-old man underwent MMS for the treatment of a nodular basal cell carcinoma (BCC) on the left nasal tip. He reported a history of tobacco use. After two stages, reconstruction with a nasalis sling flap was performed. The final defect size was 1.0 cm by 0.9 cm. Two days after surgery, the patient reported a darkening of the distal portion of the flap. He reported the use of a CPAP machine after surgery with the pressure of the machine line directly superior to the flap, with complete flap necrosis noted at 14 days. His necrosis appeared as a yellow-crusted plaque on the left nasal tip, with mild erythema of the skin surrounding the necrosis. In terms of intervention, we discussed the best course of action would be to allow continued healing through secondary intention, which would likely take two to three months to fully complete. We discussed continuing diligent wound care during this period. A 10-day course of cefuroxime 250 mg twice a day was started. We offered the patient to follow up with our Mohs service in three months and with plastic surgery for further evaluation. The patient's flap defect continued to heal by secondary intention, and one year later, at his most recent follow-up with us, his nose had healed very well.

Patient 2

A 65-year-old man underwent Mohs surgery for the treatment of a BCC on the nasal dorsum. His medical history was non-contributory. After one stage of MMS, the final defect size was 1.0 cm by 0.8 cm, and the surgical defect was closed with a complex layered repair under minimal tension. Seven days after surgery, he returned to the clinic due to complete wound dehiscence. He reported the use of his CPAP device during the postop period. His wound dehiscence appeared as a yellow, fibrinous plaque with jagged borders on the left nasal tip, with slight erythema of the surrounding skin of the plaque on the nose. The patient was reassured that the surgical site was healing well but would now have to heal via secondary intention. He was advised to keep the area covered with petrolatum ointment and bandaged until it healed. Upon re-evaluation, he was found to have a gradual filling of his dehiscence defect. He had excellent improvement and color match of the site, was very pleased with the results to date, and declined dermabrasion. At his most recent follow-up with us, his nose had healed very well.

Patient 3

**Figure 1:**
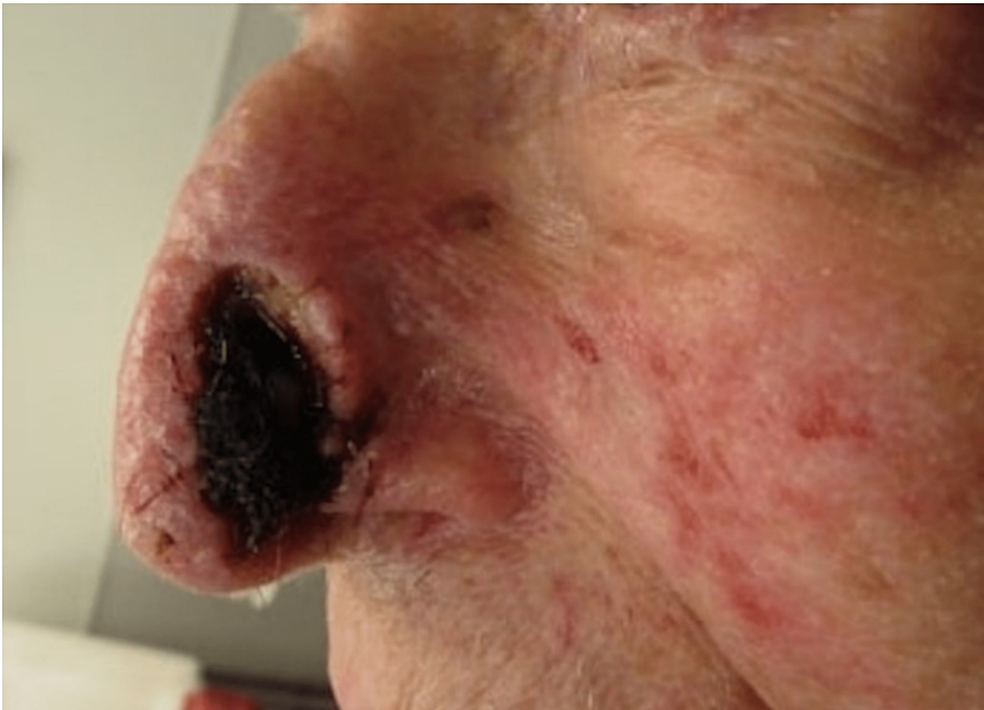
Necrosis of the nasalis sling flap

A 90-year-old man underwent Mohs surgery for treatment of a BCC on the left nasal sidewall and alar rim. He was taking apixaban. After one stage of MMS, the final defect size was 2.0 cm by 1.2 cm, and the surgical defect was closed with a full-thickness skin graft. Five months later, the patient returned with significant alar notching due to graft failure, and revision surgery with a nasalis sling flap and cartilage graft was attempted. Three weeks later, the patient returned due to necrosis of the flap (Figure 1). He reported that he wore a CPAP machine during the postoperative period of both surgeries. His necrosis appeared as a black crusted plaque on his left nasal sidewall. The superficial eschar was debrided with scissors, and the wound was dressed with petrolatum ointment, non-adherent dressing, and tape. He was brought back one week later for follow-up and offered secondary intention healing versus composite full thickness skin graft (FTSG); he opted for the latter. Cartilage to support the free margin of the defect was harvested from the left tragus. FTSG was harvested from the left preauricular cheek donor site. The FTSG was folded over the cartilage graft and secured. A bolster dressing was secured to the FTSG, which was removed one week later. During this time, the patient was referred to sleep medicine to assess if he could temporarily avoid using his CPAP device for the next several weeks during this critical stage of healing. On a follow-up visit one month later, the site appeared completely healed.

## Discussion

Knowing a patient's past medical history, such as their use of tobacco, risk of postoperative wound infection (i.e., immunosuppression), or risk of hematoma formation (i.e., blood thinner use) helps guide repair choice and postoperative care to reduce the risk of complications [1-5]. Herein, we report three cases of nasal repair failure that were associated with the postoperative use of a CPAP machine. Our experience serves to remind dermatologic surgeons of the importance of a thorough medical history to help assess a patient's risk for postoperative complications.

A prior case series noted two cases of nasal bridge skin necrosis secondary to pressure from the use of CPAP face masks [4]. The authors speculate that skin necrosis occurred from poor skin perfusion due to pressure necrosis due to the CPAP itself, with hypotension leading to local tissue anoxia [4]. In Case 1 and Case 3 of our study, the pressure of the CPAP machines likely reduced the blood flow to the distal flap inducing necrosis. In Case 2, the pressure of the mask likely reduced the blood flow to the distal nose but also may have contributed mechanically to suture rupture and subsequent wound dehiscence.

The repairs were chosen and performed with the appropriate surgical technique to counter the presence of known risk factors. For Case 1, a nasalis sling flap is a robust flap that would not be expected to show such complete necrosis even with the presence of smoking, which is known to reduce tissue survival and cause poor wound healing [2]. With Case 2, the primary closure was under minimal tension after wide-undermining, and the patient had no other risk factors. In Case 3, the necrosis started approximately three weeks after surgery, which makes apixaban-induced acute bleeding an unlikely culprit of flap necrosis. Thus, the most likely primary factor in the failure of these was thought to be CPAP machine use.

To prevent the failure of nasal defect closures from the use of a CPAP machine, we now ask patients to consider adjusting their use of the device in the immediate postoperative period. Avoidance of CPAP use is most beneficial in the first 72 hours when the blood supply is most tenuous, but complete avoidance may result in hypoxia and sleep issues. Alternatively, a soft wedge may be used to cushion the bridge of the nose from the apex of the mask [5]. Additionally, studies in neonates on CPAP showed that nasal silicone shields significantly reduced rates of nasal injuries, such as columella necrosis, suggesting that perhaps a similar shield could be used in adults [4].

## Conclusions

In conclusion, our cases illustrate the need to inquire if MMS patients are using any medical equipment, such as a CPAP machine, near the site of their Mohs procedure, in an effort to prevent undue postoperative pressure on the defect and subsequent ischemia.
